# An Induced Hypersensitive-Like Response Limits Expression of Foreign Peptides via a Recombinant TMV-Based Vector in a Susceptible Tobacco

**DOI:** 10.1371/journal.pone.0015087

**Published:** 2010-11-29

**Authors:** Mangmang Li, Ping Li, Rentao Song, Zhengkai Xu

**Affiliations:** 1 Institute of Plant Physiology and Ecology, Shanghai Institutes for Biological Sciences, The Chinese Academy of Sciences, Shanghai, China; 2 Shanghai Key Laboratory of Bio-Energy Crops, School of Life Sciences, Shanghai University, Shanghai, China; Institut Pasteur, France

## Abstract

**Background:**

By using tobacco mosaic virus (TMV)-based vectors, foreign epitopes of the VP1 protein from food-and-month disease virus (FMDV) could be fused near to the C-terminus of the TMV coat protein (CP) and expressed at high levels in susceptible tobacco plants. Previously, we have shown that the recombinant TMV vaccines displaying FMDV VP1 epitopes could generate protection in guinea pigs and swine against the FMDV challenge. Recently, some recombinant TMV, such as TMVFN20 that contains an epitope FN20 from the FMDV VP1, were found to induce local necrotic lesions (LNL) on the inoculated leaves of a susceptible tobacco, *Nicotiana tabacum* Samsun nn. This hypersensitive-like response (HLR) blocked amplification of recombinant TMVFN20 in tobacco and limited the utility of recombinant TMV vaccines against FMDV.

**Methodology/Principal Findings:**

Here we investigate the molecular mechanism of the HLR in the susceptible Samsun nn. Histochemical staining analyses show that these LNL are similar to those induced in a resistant tobacco Samsun NN inoculated with wild type (wt) TMV. The recombinant CP subunits are specifically related to the HLR. Interestingly, this HLR in Samsun nn (lacking the *N*/*N′*-gene) was able to be induced by the recombinant TMV at both 25°C and 33°C, whereas the hypersensitive response (HR) in the resistant tobacco plants induced by wt TMV through the *N*/*N′*-gene pathways only at a permissive temperature (below 30°C). Furthermore, we reported for the first time that some of defense response (DR)-related genes in tobacco were transcriptionally upregulated during HLR.

**Conclusions:**

Unlike HR, HLR is induced in the susceptible tobacco through *N*/*N′*-gene independent pathways. Induction of the HLR is associated with the expression of the recombinant CP subunits and upregulation of the DR-related genes.

## Introduction

Tobacco mosaic virus (TMV) is a plus sense single stranded RNA virus that infects plants of the family *Solanaceae*. In a susceptible host, *Nicotiana tabacum* Samsun nn, systemic infection by wild-type (wt) TMV leads to a high accumulation of TMV coat protein (CP) [1]. Due to the high output of TMV CP and facile extraction of viral particles from systemically infected plants, TMV has been intensively utilized as an expression vector to synthesize commercial foreign peptides in tobacco [2]. So far, there are several successful examples of recombinant TMVs expressing an epitope fused to the C-terminus of TMV CP in tobacco and showing enhanced immunogenicity in animals [3], [4], [5], [6], [7], [8], [9].

Foot-and-mouth disease virus (FMDV) is a viral pathogen that causes a severe epidemic of foot-and-mouth disease. Various vaccine products were developed in an attempt to protect the animals from the FMDV infection. In our laboratory we have utilized the TMV-based vector to express two B-cell epitopes (F11 and F14) of the VP1 protein from FMDV in tobacco plants for the purposes of new vaccines against FMDV [5], [9]. The resulting recombinant TMVF11 and TMVF14 infected Samsun nn systematically and were able to produce considerable yield of recombinant TMV particles. Animal tests demonstrated that the vaccines prepared from TMVF11 and TMVF14 viral particles generated strong protection in guinea pigs and swine against the FMDV challenge [5], [9].

Based upon the above results, a T-cell epitope FN20 in FMDV VP1 was selected to develop a new FMDV vaccine using the TMV-based vector. However, instead of the systemic mosaic symptoms appearing on leaves infected by TMVF11 or TMVF14 1-2 weeks after inoculation [9], local necrotic lesions (LNL) were observed on inoculated leaves of Samsun nn at 4 days post inoculation (dpi) with TMVFN20 [10]. Similarly, we also noticed that some recombinant TMV constructs designed for fusion expression of certain foreign peptides have been reported to significantly affect the infectability of the recombinant TMVs and thus the yield of the recombinant CP subunits in susceptible tobacco, due to the alterations of the recombinant CP subunits in hydrophobicity (for example TMVSC1754) [10], isoelectric point/charge value [11], or insertion of cysteine residue(s) [12]. The LNL symptoms were also observed in different susceptible tobacco hosts, such as Samsun nn and Xanthi nc inoculated by the recombinant TMVs encoding cysteine-containing or hydrophobic foreign peptides [10], [11], [13], or *N. benthamiana* inoculated by the recombinant TMVs with a insertion of an entire foreign protein [5], [11], [13], [14], [15].

Our similar observations with recombinant TMVFN20 and TMVSC1754 [10] on Samsun nn leaves allowed us to study the mechanisms of some unknown resistance pathways existing in so-called susceptible tobacco hosts that could lead us to improve the technology of expressing more foreign peptides by the TMV-based vector. In the present study, we aim to characterize the viral and host factors related to the hypersensitive-like response (HLR) that induces the LNL in susceptible tobacco Samsun nn. This HLR is specifically associated with the expression of the recombinant CP subunits. During the process of HLR, the defense response (DR)-related genes are greatly induced in inoculated tobacco plants. Our findings demonstrate that the HLR is induced in the susceptible tobacco through *N*/*N′*-gene independent pathways.

## Results and Discussion

### Histochemical studies of the LNL in Samsun nn

Besides TMVFN20, another recombinant TMVSC1754 was introduced in this study as a parallel control to better understand the HLR occurring in susceptible tobacco plants. Unlike TMVFN20, TMVSC1754 has been reported to be related to the necrotic response in Samsun nn [10] due to the fact that the fused peptide SC1754 has a transmembrane domain [16].

**Figure 1:**
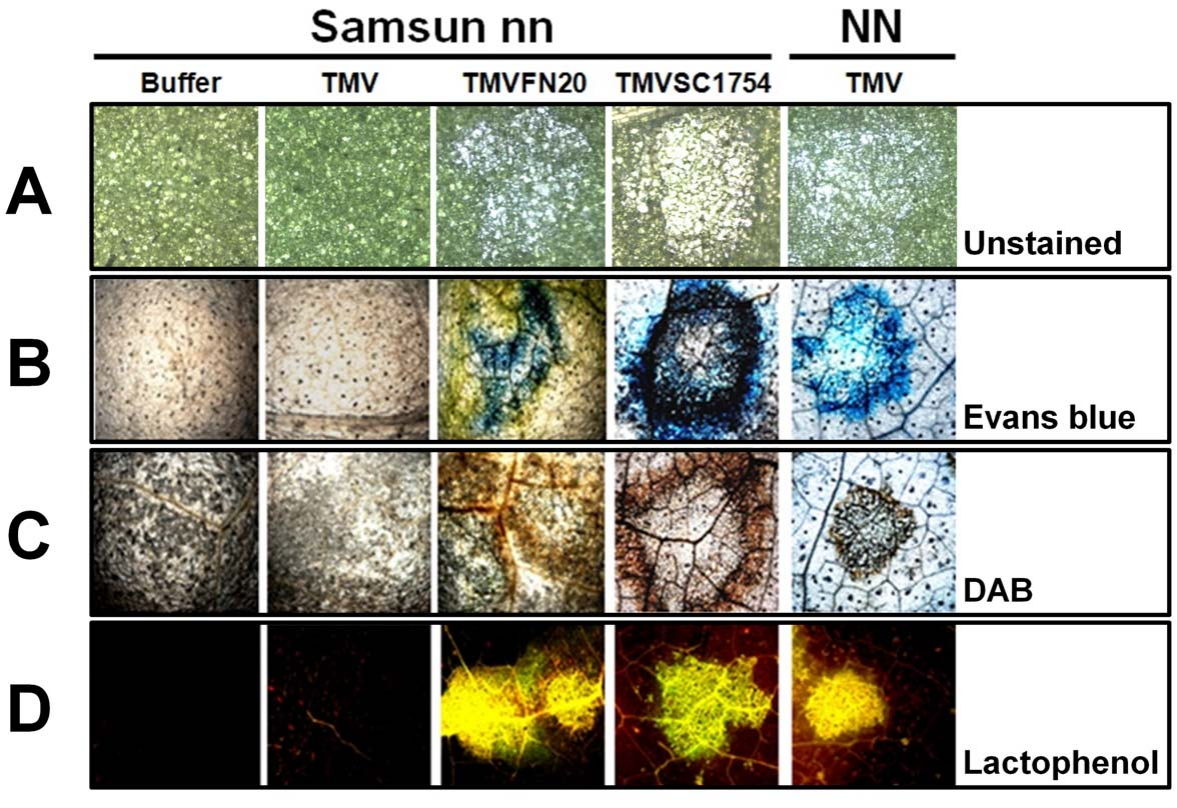
Histochemical analyses of the LNL in Samsun plants. *Nicotiana tabacum* Samsun nn were cultivated at 25°C and inoculated with infection buffer, wt TMV, TMVFN20, and TMVSC1754, respectively. At 4 dpi, necrotic lesions induced on the inoculated leaves were photographed (A) and analyzed by histochemical staining assays. Evans blue was used for staining of dead tissue (B), DAB for detection of H_2_O_2_ (C), and autofluorescence for phenolic compounds (D). HR lesions induced by wt TMV on an inoculated leaf of Samsun NN was tested in parallel.

As expected, the LNL were observed on the inoculated leaves of Samsun nn infected by TMVFN20 or TMVSC1754 at 4 dpi (Figure 1A). The HLR lesions were first monitored in histological alterations to investigate the metabolic status of host cells undergoing HLR. In parallel, hypersensitive response (HR) lesion induced by wt TMV in a resistant tobacco Samsun NN (harboring a resistance gene *N*) (Figure 1A) was also tested. The HLR induced by both TMVFN20 and TMVSC1754 resulted in irreversible cell death as evidenced by Evans blue staining [17], consistent with the observation of HR lesion (Figure 1B).

It has been reported that plant cells undergoing HR accumulated active oxygen species and autofluorescent phenolic compounds/phytoalexins inside and around necrotic lesions [18], [19]. Accumulation of these two compounds was also observed at the sites of the recombinant TMV-induced LNL (Figure 1C and D). No obvious differences in the generation of H_2_O_2_ and autofluorescence of phenolic compounds were detected between typical HR lesions and HLR lesions (Figure 1C and D). Our results suggest that the HLR induced by different recombinant TMVs undergoes similar biochemical and histological changes in susceptible tobacco, as HR does in resistant tobacco.

### The recombinant CP subunits are specifically related to HLR

**Figure 2:**
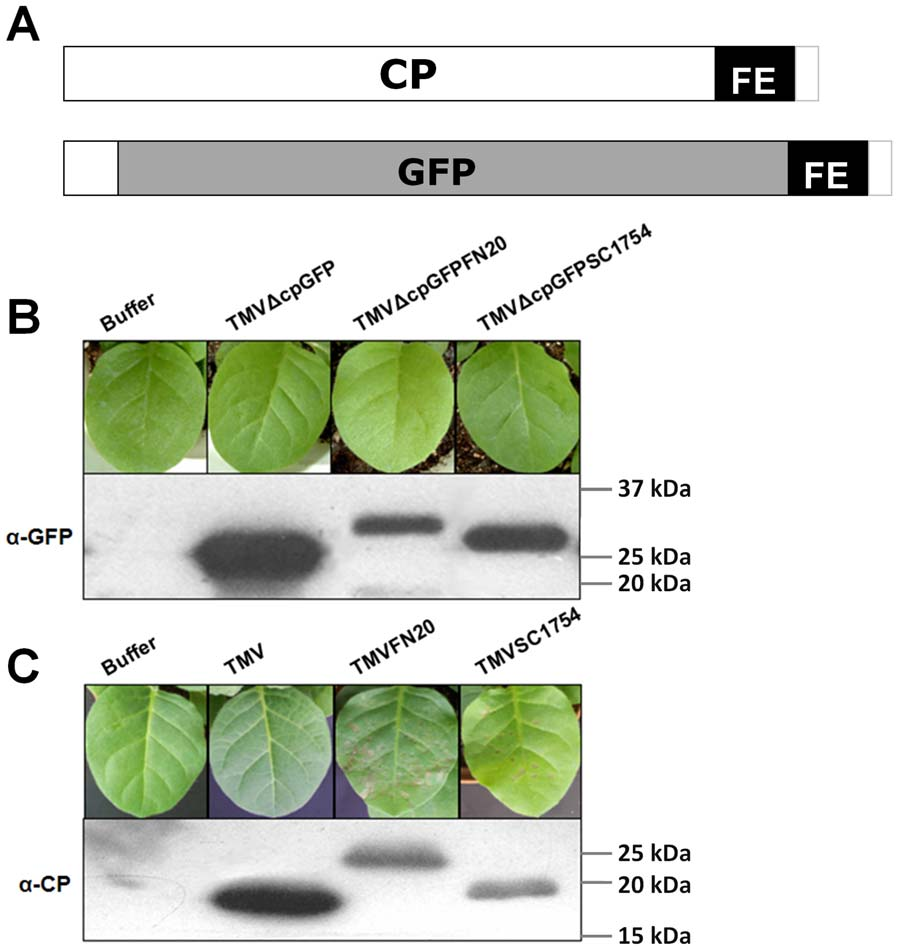
Recombinant CPs are related to the HLR in Samsun nn. (A) Structure diagrams of recombinant TMV CP and GFP proteins. Foreign peptides were fused in frame to the site S^154^-G^155^ near the C-terminus of TMV CP to generate recombinant CP proteins. GFP was inserted *in frame* downstream of the N-terminal 18 amino acid residues to replace CP. Foreign peptides were then fused to the C-terminus of GFP to generate recombinant GFP proteins. CP, coat protein. GFP, Green fluorescent protein. FE, foreign epitope. Total proteins were extracted from tobacco seedlings at 4 dpi with wt TMV, TMVΔcpGFP, TMVΔcpGFPFN20, TMVΔcpGFPSC1754 (B), TMVFN20 and TMVSC1754 (C) at 25°C, respectively. Expressed recombinant proteins were separated on 12% SDS-PAGE and visualized by using anti-GFP antibody or anti-CP antibody in western analysis. A protein standard marker (Precision Plus Protein™ standards, Bio-Rad, U.S.A.) was used to measure the sizes of recombinant proteins. Phenotypes of inoculated leaves of Samsun nn were photographed at the same time. Infection buffer was used as negative control.

To identify whether expression of the CP subunits was required for inducing HLR in Samsun nn, the main coding sequence of the CP gene in wt TMV or recombinant TMVs were replaced by the green fluorescent protein (GFP) gene to generate TMVΔcpGFP, TMVΔcpGFPFN20 and TMVΔcpGFPSC1754, respectively (Figure 2A). The 5′-terminal 54 nucleotides of the CP gene were not removed to maintain the full activity of the subgenomic CP promoter [20].

At 4 dpi with each recombinant virus, the recombinant GFP protein in the inoculated leaves of Samsun nn was detected by western blotting analysis using a rabbit antibody to GFP (Figure 2B). However, neither TMVΔcpGFPFN20 nor TMVΔcpGFPSC1754 caused LNL on inoculated leaves at 4 dpi (Figure 2B). Furthermore, no obvious phenotype was observed until 14 dpi (data not shown). As expected, TMVFN20 and TMVSC1754 induced LNL on inoculated leaves of Samsun nn at 4 dpi (Figure 2C). A rabbit antibody to TMV CP [10] was also able to detect the expression of recombinant CPFN20 and CPSC1754 at the same time (Figure 2C). The viral cDNAs from all of the recombinant TMVs were sequenced to confirm that no mutations were generated during virus replication (data not shown). This result suggests that the foreign epitope FN20 or peptide SC1754 would induce the HLR in Samsun nn only when fused to the CP subunit.

### Recombinant TMVs induced HLR via *N*/*N′*-gene independent pathways

The local necrotic response or hypersensitive response to the TMV infection was commonly seen in the resistant *N. tabacum* plants harboring the resistance gene *N* (or *N′*). HR is elicited through the specific interaction of the *N* gene product with the TMV 126-kDa replicase [21], or of the *N′* gene product with the TMV CP [22], [23].

**Figure 3:**
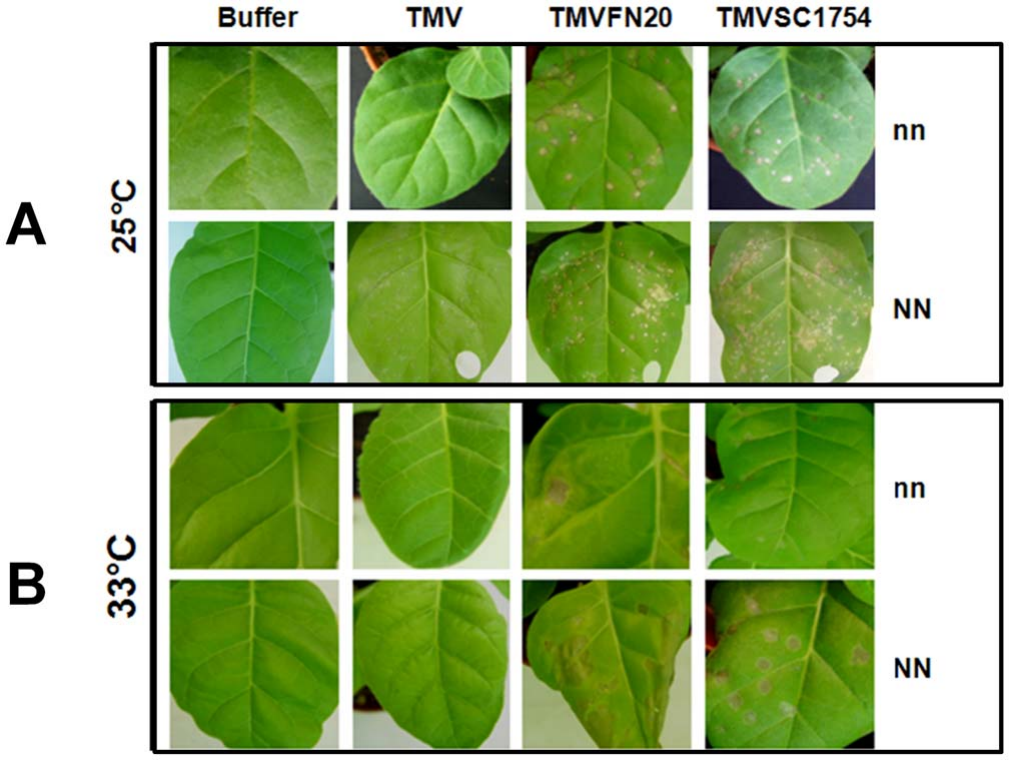
Symptoms of the HLR at different temperatures. *Nicotiana tabacum* Samsun nn and Samsun NN were cultivated in a growth chamber at 25°C (A) or 33°C (B) with a 16 h light/8 h dark photo cycle. *In vitro* transcripts of wt TMV and recombinant TMVFN20 or TMVSC1754 were rub-inoculated onto maturely detached leaves of 6-week-old tobacco seedlings. The inoculated plants were continuously cultured at 25°C or 33°C for 4 days prior to photography. Infection buffer alone was used as a negative control.

Theoretically, the TMVFN20 or TMVSC1754-induced HLR in Samsun nn was not resulted from the function of the *N* or *N′* gene (Figure 3A). To further rule out the possible function of the *N* or *N′* gene, the resistant Samsun NN was inoculated with TMVFN20, TMVSC1754 or wt TMV and the inoculated plants were incubated at 25°C or 33°C. It has been known that the *N*-mediated HR can only be properly induced by TMV in Samsun NN at a permissive temperature (below 30°C) [21]. When the temperature is raised above 30°C, Samsun NN becomes susceptible to the TMV infection. As shown in Figure 3B, the LNL appeared only on the Samsun NN leaves inoculated with TMVFN20 or TMVSC1754 but not wt TMV at 33°C, whereas all of recombinant TMVs and wt TMV induced the LNL on the inoculated leaves of Samsun NN at 25°C (Figure 3A). Similar local necrotic response was also induced in Samsun nn at 33°C by recombinant TMVs but not wt TMV (Figure 3B). We thus conclude that the mechanism resulted in the HLR induced by recombinant TMVs is independent of the *N/N′* gene. In addition, more LNL were induced by recombinant TMVs in Samsun NN than in Samsun nn at 25°C probably due to a synergistic action of HLR and HR (Figure 3A). In both Samsun nn and Samsun NN, however, the HLR lesions are larger in size and fewer in number at 33°C than that at 25°C (Figure 3), suggesting that signaling of HLR was slower at the higher temperature. However, no more LNL were induced on the inoculated leaves of Samsun nn and Samsun NN at both 25°C and 33°C till 14 dpi with either TMVFN20 or TMVSC1754, compared to that at 6 dpi (data not shown). This suggests that elicitation of HLR is completed prior to 6 dpi at either 25°C or 33°C. Furthermore, HLR could still be induced by recombinant TMVs in Samsun nn at two extreme temperatures (16°C and 36°C, data not shown), indicating that elicitation of HLR is stable in a larger temperature range compared to that of HR.

### Evaluation of DR-related genes expressed during HLR

HR is a mechanism of DR that is triggered by host-pathogen recognition resulting in regulation of gene expression as well as modulating protein interactions [24], [25]. DR-related genes could be roughly classified into three functional groups: PR protein genes, secondary metabolites and oxidative burst-related genes, and programmed cell death-related genes (Table 1). Some of DR-related genes were induced during HR [26], [27], [28], [29], [30], [31].

**Table 1:** Defense response-related genes in this study.

Classification	GenBank Accession #	Annotation
**First group: Pathogenesis-related protein genes**		
*PR-1a* (*pathogenesis-related protein-1a*)	D90196	Unknown [32]
*PR-1b* (*pathogenesis-related protein-1b*)	D90197	Unknown [32]
*PR-1c* (*pathogenesis-related protein-1c*)	X17681	Unknown [32]
*PR-2d* (*acidic β-1,3-glucanase*)	X69794	Secreted in the extracellular spaces of the plant and is stress dependent [40]
*PR-2e* (*basic β-1,3-glucanase*)	M59442	Associated with intracellularly mediated defense response within the central vacuole of the plant cell [40]
*PR-5c* (*osmotin*)	X61679	Accelerates adaptation of plant cells to osmotic stress [41]
*PR-6* (*PINII, proteinase inhibitor ã?/emph>)*	Z29537	Type II serine protease inhibitor [42]
*PR-8* (*basic class III chitinase*)	Z11564	Bifunctional chitinase/lysozyme in vacuole of the plant cell [30]
**Second group: Second metabolites and oxidative burst-related genes**		
*HMGR* (*3-hydroxy-methylglutaryl CoA reductase*)	AF004233	Membrane-bound enzyme involved in sterol biosynthesis [33]
**Third group: Programmed cell death-related genes**		
*HIN1* (*harpin-induced 1*)	AB091429	Associated with leaf senescence [27]

**Figure 4:**
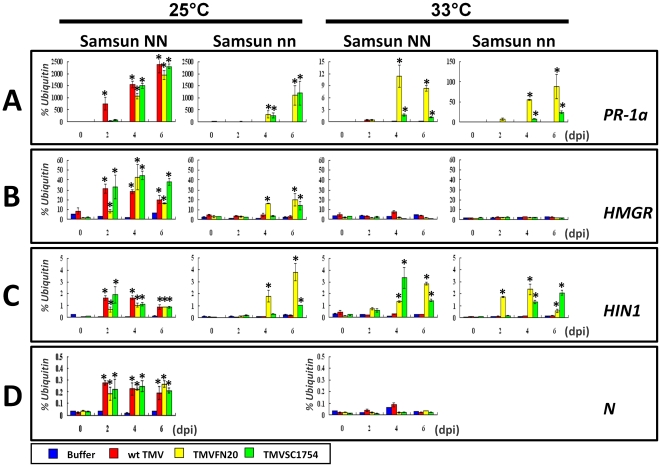
Expression evaluation of the DR-related genes during HLR. Seedlings of Samsun nn and Samsun NN were inoculated with *in vitro* transcripts of wt TMV (Red), TMVFN20 (Yellow) and TMVSC1754 (Green) as mentioned in Experimental Procedures. Infection buffer (Blue) was used as negative control. Inoculated seedlings were incubated at 25°C and 33°C respectively and sampled for real-time PCR assay at 0, 2, 4, 6 dpi. Data are shown as the mean of at least two biologically repeated experiments, and the error bar is the standard error (SE). Expression values of each gene are presented as the percentage of the reference gene *ubiquitin*. At 0 dpi, average expression values of the genes in Samsun plants at 25°C are 0.45±0.18 for *PR-1a* (A), 3.16±0.68 for *HMGR* (B), 0.05±0.01 for *HIN1* (C), and 0.03±0.00 for *N* (D) in terms of mean ± SE percent of *ubiquitin*, respectively. Signals of each gene at different time points (2, 4, 6 dpi) were compared to that at the initial time point (0 dpi) by t test, *p<0.01.

To characterize HLR at a molecular level, expression profiles of DR-related genes were examined by quantitative PCR analysis. Expression of the resistance gene *N* in Samsun NN was also tested in parallel. Samsun nn or Samsun NN was incubated at 25°C or 33°C for 6 days after inoculation with TMVFN20, TMVSC1754 or wt TMV. RNA samples were extracted from the inoculated leaves at 0, 2, 4 and 6 dpi for reverse transcription and Real-time PCR analyses using gene specific primers (Table S1). The results were summarized in Figure 4 and Figure S1.

*PR-1a* gene (Figure 4A): The *PR-1a* gene belongs to the family of PR protein genes [32]. In TMVFN20- or TMVSC1754-inoculated leaves of Samsun nn, transcription of *PR-1a* was greatly upregulated at 4 dpi at 25°C (Figure 4A). In Samsun NN, *PR-1a* was also induced at the same time point at 25°C (Figure 4A). Unlike in Samsun nn, expression of *PR-1a* at 25°C was induced in wt TMV infected-Samsun NN at 2 dpi, consistent with previously reported data [32]. As expected, no increase of *PR-1a* expression was detected in either Samsun nn or NN until 6 dpi with wt TMV at 33°C. However, the extent of transcriptional upregulation of *PR-1a* induced by recombinant TMVs in both Samsun nn and NN was greatly reduced at 33°C (Figure 4A). This result strongly suggests a function of *PR-1a* in HLR. Future work will address the molecular mechanism of PR-1a protein in HLR.

*3-hydroxy-methylglutaryl CoA reductase* gene (*HMGR*) (Figure 4B): The *HMGR* gene has been identified as a membrane-bound enzyme involved in sterol biosynthesis [33]. At 25°C, transcription of *HMGR* was significantly induced in Samsun nn at 4 dpi with TMVFN20 and at 6 dpi with TMVSC1754, respectively (Figure 4B). However, it was upregulated at 2 dpi in Samsun NN inoculated with wt TMV, TMVFN20 or TMVSC1754 at 25°C (Figure 4B). Interestingly, it was not induced by recombinant TMVs at 33°C in either Samsun nn or Samsun NN, even though HLR was induced on inoculated leaves (Figure 3). Considering the slower elicitation of HLR at 33°C (Figure 3B), signaling involved the *HMGR* gene induction may not play a role in the elicitation of HLR at 33°C.

*Harpin-induce 1* gene (*HIN1*) (Figure 4C): The *HIN1* gene is directly related to leaf senescence [27]. Transcription of *HIN1* was greatly upregulated by inoculation of recombinant TMVs in both Samsun nn and Samsun NN at two different temperatures (25°C and 33°C) (Figure 4C). Compared to wt TMV inoculation in Samsun NN at 25°C, both of recombinant TMVs induced upregulation of *HIN1* to a similar extent (Figure 4C), suggesting a critical role of *HIN1* in HLR.

*N* gene (Figure 4D): As expected, the known resistance gene *N* was significantly induced by wt TMV in Samsun NN at 25°C, but not at the intolerant temperature 33°C (Figure 4D). Moreover, inoculation of recombinant TMVs also induced the transcription of *N* in Samsun NN only at 25°C (Figure 4D). This result indicates that the typical HR may be simultaneously elicited by recombinant TMVs due to the interaction of viral 126 kDa replicase and host N protein at 25°C [21]. However, induction of HLR was induced at both 25°C and 33°C independent of *N*. Hence the HLR observed in Samsun nn and Samsun NN inoculated by recombinant TMVs may be elicited by an unknown host resistance protein.

To extend our understanding of expression of the DR-related genes in HLR, transcriptional alterations of 7 more DR-related genes were investigated by real-time PCR. At 25°C, all of these DR-related genes were induced by infection of TMVFN20 or TMVSC1754 in Samsun nn or by infection of recombinant TMVs and wt TMV in Samsun NN (Figure S1). However, most of DR-related genes except genes *PR-1b*, *PR-1c* and *PR-6* were only slightly induced or not induced at all in both Samsun nn and Samsun NN at 33°C (Figure S1). This indicates that fewer DR-related genes were involved in HLR at 33°C than those at 25°C. It may partially explain why elicitation of HLR was slower at 33°C than at 25°C (Figure 3). Like *HIN1*, *PR-1b, c* and *PR-6* were upregulated at 4 dpi at 33°C (Figure S1). Furthermore, two different recombinant TMVs resulted in significantly different upregulation of some DR-related genes, such as *HIN1* in Figure 4C and *PR-1c* in Figure S1. Taken together, our results suggest that different foreign peptides fused to TMV CP might elicit the HLR via variable pathways.

Our results show that fusion of TMV CP with different foreign peptides induced a similar HLR at both the biochemical and molecular level in *N. tabacum* Samsun. Compared to typical HR in Samsun NN, HLR is a type of resistance response against recombinant TMVs covering a wider temperature range in both susceptible and resistant tobacco plants. Currently, researchers are experiencing many difficulties in trying to express foreign peptides or proteins in substantial amounts in tobacco plants using TMV-based vectors [2]. This is mainly due to the host resistance response that occurs in susceptible tobacco. However, little is known about the molecular mechanism of HLR [5], [11], [13], [14], [15]. So far, only four examples of direct recognition of pathogen effectors by host resistance proteins have been identified in plants [34], [35], [36], [37]. Recently, a chloroplastic protein NRIP1 was reported to interact with both an innate immune receptor N (*N* gene product) and a viral effector p50 of the TMV 126 kDa replicase in resistant tobacco to elicit the *N*-mediated HR [38]. However, *N* gene does not contributed to induction of HLR in susceptible tobacco. Our data suggested that the HLR is controlled by a mechanism different from the HR associated with N protein. Obviously, the HLR has greatly limited the application of TMV-based expression vectors in vaccine research.

To avoid this restriction on foreign epitope expression via recombinant TMV vector in tobacco, a novel peptide-display system in which the RNA genome of Semliki Forest virus (SFV) was trans-encapsidated *in vitro* by purified TMV CP has been developed [39]. The assembled SFV/TMV CP capsid was able to display foreign epitopes on its surface through genetic fusions or chemical conjugation. This new system could be a good complement for genetically engineered TMV being used as nanoparticle vaccines, although more improvements are needed prior to mass production [2]. Our work has shown that HLR was elicited in a similar fashion as HR at both the histochemical and biochemical levels, suggesting that a certain host protein may be responsible for the HLR phenotype. In this study, some DR-related genes were demonstrated to be associated with the HLR. Further work such as identification of the unknown host resistance gene or important genes involved in HLR signaling is merited. Gene knockout of these critical genes could be a tool to avoid HLR in susceptible tobacco, leading to an additional strategy for attempts at efficient expression of foreign peptides or proteins that have failed by current methods.

In summary, our study shows that the recombinant TMVFN20 or TMVSC1754 induces a HLR through *N*/*N′*-gene independent pathways in the susceptible tobacco Samsun nn. The recombinant CP subunits are specifically involved in the HLR. Given that some DR-related genes are greatly induced during HLR, our findings provide new insight into the local resistance response against recombinant TMVs in susceptible tobacco and could be helpful in the expression of foreign epitopes through TMV-based vectors in tobacco.

## Materials and Methods

### Plant materials and treatments

Tobacco plants (*Nicotiana tabacum* Samsun nn and Samsun NN) were cultivated in a growth chamber at 25°C with a 16 h light/8 h dark photo cycle. *In vitro* transcription of the recombinant TMV or wt TMV plasmid was rub-inoculated with infection buffer (50 mM phosphate buffered saline, pH 7.0, and 1 mM EDTA) onto maturely detached leaves of 6-week-old tobacco seedlings as mentioned previously [10]. Infection buffer was used alone as a control. Infection phenotype was observed by incubating the inoculated plants for 6 days at 25°C or 33°C, respectively.

### Plasmid construction

Plasmids pTMVFN20 and pTMVSC1754 were constructed from pTMV by inserting foreign peptide coding sequences of FN20 (ETQVQRRQHTDVSFILDRFV) or SC1754 (FFFVSYIIISFLVVVNMY) between codons of Thr^153^ and Trp^152^ in the TMV CP as described previously [10]. To construct plasmid pTMVΔcpGFP, a multiple cloning site (*Kpn*I/*Xho*I/*Eco*RV/*Not*I) was firstly introduced downstream the stop codon of the CP gene in pTMV to generate a new plasmid, pTMV-KXEN. pTMVΔcp was then generated from pTMV-KXEN by partially deleting the CP gene from downstream +55 bp of the start codon of CP. The green fluorescent protein (GFP) gene was amplified by using primers 5′-AAGGATATCATGAGTAAAGGAGAAGAAC-3′ and 5′-TTTTCCTTTTGCGGCCGCTCATTTGTAGAGCTCATCC-3′ from pGFPuv (Clontech, U.S.A.) and sub-cloned into *EcoR*V/*Not*I sites in pTMVΔcp to obtain pTMVΔcpGFP. Foreign peptides FN20 and SC1754 were then inserted upstream the stop codon of the GFP gene in pTMVΔcpGFP to construct pTMVΔcpGFPFN20 and pTMVΔcpGFPSC1754.

### Histochemical analysis

Infected leaf tissues of *N. tabacum* Samsun nn and Samsun NN were processed for histochemical analysis and observed by microscopy. Evans blue staining was used to indicate cell death as described previously [17]. Briefly, leaf tissues were incubated in 0.25% Evans blue for 15 min and then boiled in 96% ethanol for 5 min to remove chlorophylls. Cell death was observed under a light microscope. The presence of H_2_O_2_ was detected as described previously [19]. Briefly, leaf tissues were incubated in 1 mg/ml 3,3′-Diaminobenzidine-HCl (DAB-HCl, pH 3.8, Sigma, U.S.A.) for 6–8 h at room temperature in dark, and then boiled in 96% ethanol to remove chlorophylls. The accumulation of H_2_O_2_ was detected under a light microscope. Formation of phenolic compounds was detected as described previously [18]. Briefly, leaf tissues were boiled in lactophenol buffer (phenol/lactic acid/glycerol, 1∶1∶1, v/v) for 2 min, and then washed successively with 50% ethanol and water. Phenolic compounds were observed for their yellow fluorescence with a UV filter under a microscope.

### Western analysis

Total proteins from 1 leaf disk (1 cm in diameter) of wt TMV-infected leaves or recombinant TMVs-induced necrotic lesions were extracted in 100 µl of SDS-sample buffer (62.5 mM Tris-Cl, pH 6.8, 2% SDS, 0.002% bromphenol blue, 10% glycerol, 2% β- mercaptoethanol) by incubating at 100°C for 5 minutes and then centrifuging at 15,000 g for 15 min. 20 µl of soluble proteins were separated by 12% SDS-PAGE. After electrophoresis, the proteins on the gel were electro-transferred onto a PVDF membrane (0.2 µm pore size, Invitrogen, U.S.A.). After being incubated in a blocking buffer (5% skim milk, 20 mM Tris-HCl, 500 mM NaCl, 0.05% Tween-20, pH 7.5) for 1 h, the PVDF membrane was incubated with rabbit anti-TMV CP serum (1∶500 dilution in the blocking buffer) or with rabbit anti-GFP serum (1∶1000 dilution, Invitrogen, U.S.A.) for 1 h, followed by goat anti-rabbit IgG conjugated to horseradish peroxidase (1∶10,000 dilution, Pierce, U.S.A.) for 1 h, and then developed with a chemiluminescence system (ECL, Amersham, U.S.A.).

### RNA isolation and quantitative RT-PCR

Total RNA was extracted from 1 leaf disk with 500 µl Trizol™ reagent according to the protocol (Invitrogen, U.S.A.). 5 µg of DNase I-treated total RNAs were used as template for reverse transcription (RT) in 20 µl volume by using SuperScript™ III First-Strand Synthesis System (Invitrogen, U.S.A.). The RT reaction was carried out by following the product manual. 1 µl of the RT product was amplified in 15 µl of real-time PCR reaction. PCR parameters were: 95°C for 10 min, 40 cycles of 95°C for 30 s, 55°C for 30 s, 72°C for 30 s. Real-time PCR was performed on a DNA engine opticon 2 system (MJ Research, U.S.A.) using the SYBR Green Real-time PCR Master Mix as mentioned in the protocol (TOYOBO, Japan). Expression level of each investigated gene was normalized by the ubiquitin mRNA. Primers were listed in Table S1.

## Supporting Information

Figure S1 **DR-related gene expression profiles in tobacco plants infected with recombinant TMVs.** 6-week-old tobacco seedlings (Samsun nn and NN) were inoculated with *in vitro* transcripts of TMV (Red), TMVFN20 (Yellow) and TMVSC1754 (Green) as mentioned in Experimental Procedures. Infection buffer was used as negative control (Blue). Infected seedlings were incubated at 25°C or 33°C and sampled for real-time PCR assay at different time points (0, 2, 4, 6 dpi) as listed on X-axis. The DR-related genes were evaluated in transcription regulation according to various virus challenges. Data are shown as the mean of at least two biologically repeated experiments, and the error bar is the standard error (SE). The expression value of each gene is presented as the percentage of the reference gene *ubiquitin*. At 0 dpi, average expression values of the genes in Samsun plants are 0.26±0.05 (*PR-1b*), 0.23±0.07 (*PR-1c*), 0.01±0.00 (*PR-2d*), 60.92±18.64 (*PR-2e*), 47.57±6.09 (*PR-5c*), 0.04±0.01 (*PR-6*), and 0.06±0.01 (*PR-8*) in terms of mean ± SE percent of *ubiquitin*, respectively. Signals of each gene at different time points (2, 4, 6 dpi) were compared to that at the initial time point (0 dpi) by t test, *p<0.01.
(DOC)

Table S1 **Primer sequences used for real-time PCR.**
(DOC)
