# Supplementary figures and images for: An Induced Hypersensitive-Like Response Limits Expression of Foreign Peptides via a Recombinant TMV-Based Vector in a Susceptible Tobacco
Source: PLoS One. 2010 Nov 29;5(11):e15087. doi: 10.1371/journal.pone.0015087 (PMC2993970; doi:10.1371/journal.pone.0015087)

Supporting Information S1


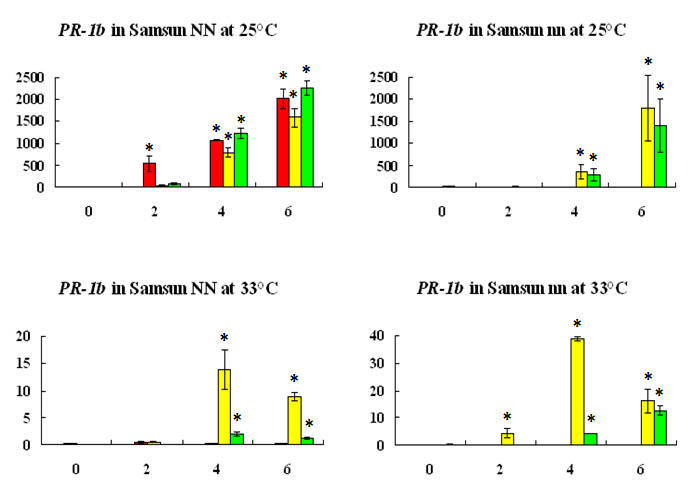

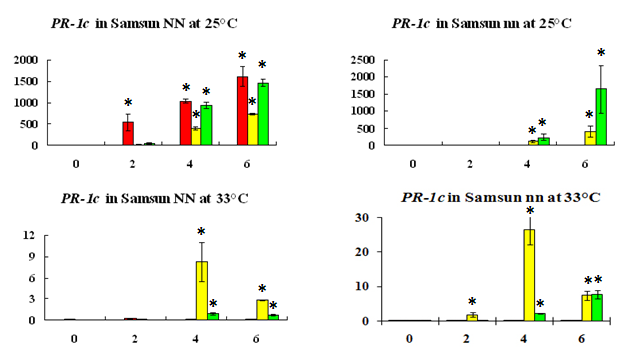

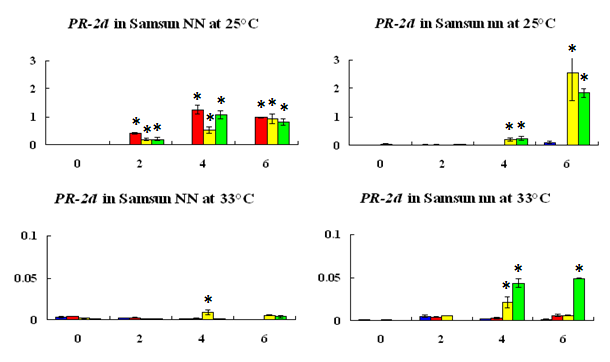

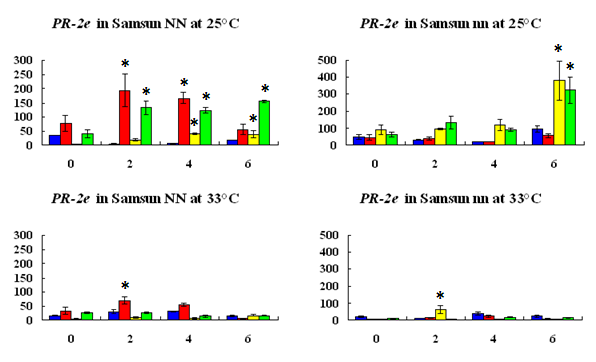

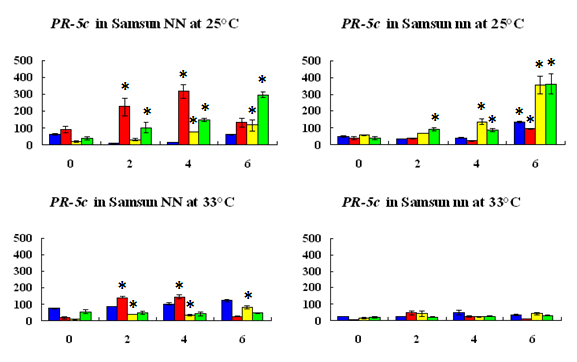

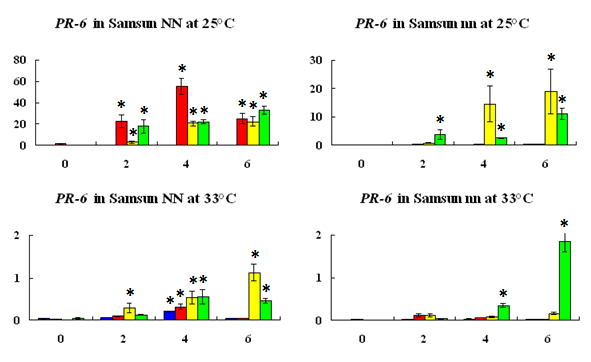

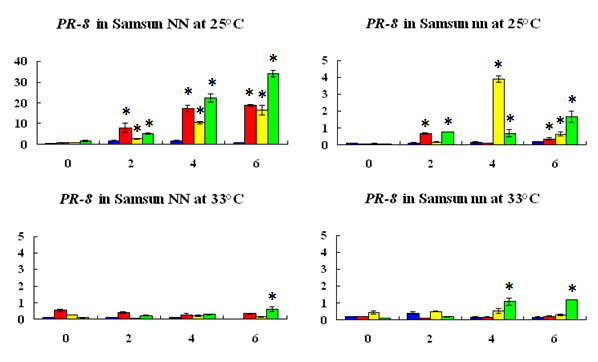

Supplement: Figure S1 — DR-related gene expression profiles in tobacco plants infected with recombinant TMVs. 6-week-old tobacco seedlings (Samsun nn and NN) were inoculated with in vitro transcripts of TMV (Red), TMVFN20 (Yellow) and TMVSC1754 (Green) as mentioned in Experimental Procedures. Infection buffer was used as negative control (Blue). Infected seedlings were incubated at 25°C or 33°C and sampled for real-time PCR assay at different time points (0, 2, 4, 6 dpi) as listed on X-axis. The DR-related genes were evaluated in transcription regulation according to various virus challenges. Data are shown as the mean of at least two biologically repeated experiments, and the error bar is the standard error (SE). The expression value of each gene is presented as the percentage of the reference gene ubiquitin. At 0 dpi, average expression values of the genes in Samsun plants are 0.26±0.05 (PR-1b), 0.23±0.07 (PR-1c), 0.01±0.00 (PR-2d), 60.92±18.64 (PR-2e), 47.57±6.09 (PR-5c), 0.04±0.01 (PR-6), and 0.06±0.01 (PR-8) in terms of mean ± SE percent of ubiquitin, respectively. Signals of each gene at different time points (2, 4, 6 dpi) were compared to that at the initial time point (0 dpi) by t test, *p<0.01. (DOC) [file pone.0015087.s001.doc]
