# Supplementary material for: An Induced Hypersensitive-Like Response Limits Expression of Foreign Peptides via a Recombinant TMV-Based Vector in a Susceptible Tobacco
Source: PLoS One. 2010 Nov 29;5(11):e15087. doi: 10.1371/journal.pone.0015087 (PMC2993970; doi:10.1371/journal.pone.0015087)
Supplement: Table S1 — Primer sequences used for real-time PCR. (DOC) [file pone.0015087.s002.doc]

Table S1. Primer sequences used for real-time PCR

| Gene | Forward primer | Reverse Primer | Position of PCR product in gene |
| --- | --- | --- | --- |
| *Ubiquitin* | ATGCAGATCTTCGTGAAGACAT | CGGGTTGACTCTTTCTGGAT | 164-363 |
| *PR-1a* | ATTGGCTGCAGATTGTAACCTCGTA | CGCCAAACCACCTGAGTATAGTGTC | 212-403 |
| *PR-1b* | TGCCCAAAACTCTCAACAAG | AGCCGTCATAAAATCGCCACT | 104-308 |
| *PR-1c* | TTGGATGCCCATAACACAGC | CACATCTCGACGGCCTTA | 582-781 |
| *PR-2d* | AAAATGGCTTTCTTGCAGC | GCATTGAAGACATTTGTT | 1776-2193 |
| *PR-2e* | TGTGTATGCTGCCCTCGAGC | TTTGGGCTGCTTGTTGGGG | 3104-3368 |
| *PR-5c* | GCGAACTTAGGGTTCCCGGA | TCCAGGCATTTCCAAGGGAA | 464-717 |
| *PR-6* | TGACCCCAAAAATCCAAACG | CGCCATCCTGACCGAAGTAG | 668-825 |
| *PR-8* | GACTTCCAGCAGCCAAGACAG | TAAACAGCACCCCTGATAGC | 718-889 |
| *HMGR* | AATAGTTGCTGGTTCTGTTCTTGCT | ATTGGATTGTCTTCCCCTCATT | 1788-1934 |
| *HIN1* | CTTCATTTTTCTTGAGCCATGCCGGA | GCAGAGGCAGCCAAAGAGACAGCTA | 68-211 |
| *N* | GTCGAAGAAGTGATGAGCAACAACA | CGACCTCCCCATGTTAAATACCCTA | 1526-1666 |
